# Exploring health seeking behavior among men who have attempted suicide - a qualitative study from Germany

**DOI:** 10.1186/s12888-025-07420-z

**Published:** 2025-09-23

**Authors:** Cora Spahn, Juliane Brüdern, Sascha Kranz, Johanna Panitz, Katarina Stengler, Christine Rummel-Kluge, Maria Strauß, Heide Glaesmer

**Affiliations:** 1 Department of Medical Psychology and Medical Sociology, Leipzig University, Leipzig, Germany; 2 Department of Psychiatry, Psychotherapy and Psychosomatics, Helios Park Hospital Leipzig, Leipzig, Germany; 3 Department of Psychiatry and Psychotherapy, Medical Faculty, Leipzig University, Leipzig, Germany; 4 Department of Psychiatry and Psychotherapy, University Leipzig Medical Center, Leipzig, Germany

**Keywords:** Suicide attempt, Men, Help-seeking, Facilitating inhibiting aspects

## Abstract

**Background:**

Men are at a significantly higher risk of dying by suicide than women. At the same time, they are less likely to seek help compared to women, alongside other risk factors. The aim of this qualitative study is to examine the facilitating and inhibiting aspects that affect help-seeking in men who have attempted suicide in order to inform gender-specific suicide prevention.

**Methods:**

Fourteen qualitative, problem-centered interviews were conducted with men who have had at least one suicide attempt in their life. Facilitating and inhibiting aspects for help-seeking were analyzed using structuring qualitative content analysis.

**Results:**

Facilitating aspects for seeking help include access to professional support, social resources, favorable psychological resources, positive experiences with the support system, and symptom burden. Inhibiting aspects include perceived inaccessibility of professional support, psychological aspects that hinder help-seeking, negative experiences with the support system, stigma/guilt and shame, and hegemonic masculinity norms.

**Conclusions:**

The findings regarding inhibiting and facilitating aspects offer various avenues to improve men’s access to psychiatric and psychosocial support systems, as well as to advance gender-specific suicide prevention at the intra-individual level (e.g., strengthening favorable psychological resources), the interpersonal level (e.g., training gatekeepers for men with few social resources), and the societal level (e.g., availability of psychosocial support).

**Supplementary Information:**

The online version contains supplementary material available at 10.1186/s12888-025-07420-z.

## Introduction

Men are at a significantly higher risk of dying by suicide than women in almost every country in the world. In Germany, the gender ratio is around 3 to 1 [1]. Several gender-specific risk factors have been discussed as possible explanations for the increased risk of suicide among men. However, evidence is limited and existing research has mainly focused on distal factors rather than underlying mechanisms or proximal risk factors. In their review, Richardson et al. [2] identified a status of being single or widowed, alcohol and/or drug abuse/dependence and a diagnosis of depression as specific risk factors in men. There is also sufficient evidence to support the assertion that men tend to use more lethal suicide means than women [3, 4].

In addition to the male-specific risk factors, the lower tendency of men to seek help in suicidal crises is of particular interest and has been shown in a number of studies [5–8]. Help-seeking is a complex process. It can be defined as ‘an adaptive coping process in which attempts are made to seek external support to deal with a mental health problem’ ( [9], p. 180).

About two-thirds of men who die by suicide have had no previous contact with the psychosocial support system [10]. Moreover, men are less likely to talk about their suicidal thoughts than women [11]. Seeking help from the private and professional support system in the event of suicidal thoughts and behavior is, however, central to suicide prevention. Men experiencing a suicidal crisis do not only tend to seek less treatment, they are also more likely to discontinue treatment or are less likely to resume it during a subsequent crisis. Spahn et al. [12] found that, compared to women, men make less use of outpatient psychiatric and psychotherapeutic treatment after an inpatient stay due to a suicide attempt or acute suicidal crisis: 52% of men do not use outpatient support services at all. Cleary´s [13] study of 52 men found that after discharge from hospital following a serious suicide attempt, 32.7% did not use psychiatric aftercare service and 20% only attended for a short time.

Seidler et al. [14] examined the continuity of mental health service use and reasons for discontinuation in 1,907 Australian men. The therapy dropout rate was 44.8% (*n* = 855), with 26.6% (*n* = 120) not returning after the first therapy session. Current depressive symptoms as well as suicidality were more common among those who had dropped out compared to the non-dropouts.

Only a few studies have dealt with male-specific barriers to seeking help during suicidal crises [15]. One key finding was that masculinity norms and stigmatization represent significant barriers to seeking help, aligning with existing research on obstacles to mental health care, particularly in the context of depression [8, 16–20]. According to Jones et al. [15], further barriers are men’s difficulty in recognizing psychological symptoms as well as clinicians’ (false) rejection of men’s support needs. Similarly, Cleary [13] cites a lack of awareness of psychiatric symptoms, an unwillingness to disclose one’s distress and a negative attitude towards seeking professional help as potential barriers. However, facilitating aspects regarding help-seeking behavior in the context of suicidal thoughts and behavior have received only limited attention in clinical practice and research [21]. Sheik et al. [21] demonstrated in a review on help-seeking behavior for mental health among boys and young men that gender-sensitive campaigns and self-compassion serve as facilitating aspects.

Given the paucity of research in this area, the present study aimed to examine inhibiting and facilitating aspects affecting help-seeking in men with lifetime suicidal behavior. The conceptual framework of the study draws on theoretical assumptions of the Help-Seeking Process Model within the context of psychological crises. In particular, it is based on a model that outlines the help-seeking process in four key stages: recognizing the issue at hand, deciding to seek assistance, and seeking, finding, and utilizing help [9]. We analyzed aspects linked to the personal environment (such as friends or family) as well as aspects associated with professional mental health care. The findings of this study are meant to bridge the existing research gap and to inform suicide prevention efforts.

## Methods

### Participants and procedure

The qualitative interviews were part of the MEN-ACCESS study, a collaborative research project conducted between March 2021 and April 2024 by Leipzig University, Medical School Berlin, and Bielefeld University. The aim of the project was to develop online tools for men at risk of suicide and their relatives in Germany. As part of the development process, qualitative interviews were conducted at Leipzig University with men who had attempted suicide at least once in their lives. At Medical School Berlin, a suicide prevention tool was developed for relatives of men at risk of suicide, following a gatekeeper approach [22]. In this context, qualitative interviews were conducted with individuals who had lost a male relative to suicide.

We employed criterion-based sampling in the selection of participants. The participants were required to be at least 18 years of age, to demonstrate a sufficiently good command of German and had to identify as male. Participants were further required to have at least one lifetime (aborted) suicide attempt and to agree with the question: “Have you ever attempted suicide with the intention of dying?” (question from the Self-injurious thoughts and behaviors interview SIT-BI [23]). Cognitive impairments and psychotic symptoms were exclusion criteria for study participation.

Participants were recruited between September 2021 and April 2023 in cooperation with two psychiatric hospitals in Leipzig, Germany. Patients who met the inclusion criteria were approached in the hospitals, and the information leaflet for the study was distributed in outpatient treatment centers, via the newsletter/website of the Leipzig Alliance Against Depression (“Buendnis gegen Depression e.V.“) or through media reports. Using these recruitment methods, a total of 15 men were recruited for the study. All individuals who met the inclusion criteria were included. One participant was excluded due to psychotic symptoms. One interview was conducted online; the others took place in person, either in an inpatient setting or at the Department of Medical Psychology and Medical Sociology at the University of Leipzig. The interviews lasted approximately two hours. All participants received information on the content and procedure of the interview, the aim of the study, data protection policies, the voluntary nature of participation and provided written informed consent. An amount of 40 euro was provided to each participant as reimbursement to cover travel and time expenses incurred through study participation.

Participants meeting the inclusion criteria and providing written informed consent were included in the study. Data collection was concluded after 14 interviews, as sufficient data saturation was reached [24]. Given the clearly defined research questions and the relatively homogeneous sample, it is expected that 14 interviews were adequate. Participants were aged between 19 and 61 years (M = 43.72, SD = 13.72), including 13 cisgender men and one transgender man. Nine of the 14 study participants (64.2%) had attempted suicide more than once and one study participant (7.2%) reported one aborted suicide attempt. All study participants made use of psychotherapeutic or psychiatric support services. The participants’ clinical characteristics are presented in Table 1.

**Table 1:** Clinical characteristics of the participants (N/A = not applicable, *= treatment by psychiatrists, **= treatment by psychotherapists)

Participant (*P*)	P01	P02	P03	P04	P05	P06	P07	P08	P09	P10	P11	P12	P13	P14
Age	56	39	60	24	38	19	61	42	39	60	27	48	53	46
Current sexual orientation	hetero	hetero	hetero	hetero	hetero	hetero	hetero	hetero	hetero	homo	hetero	hetero	hetero	hetero
Number of lifetime suicide attempts	3	1	1	2	2	several	1	2	1	3	1	3	2	2
Diagnosis/ mental disorder	Depression	Bipolar affective disorder	Depression	ADHD, Depression	Depression	Depression, personality disorder	Depression	Depression	Anxiety disorder, depression	Schizoaffective disorder with bipolar manifestations, depression	Depression	Schizophrenia	Depression	Depression, schizophrenia, adjustment disorder
Current medication (psychotropic drugs)	yes	no	yes	no	yes	yes	yes	yes	yes	yes	yes	yes	yes	no
Psychiatric hospital/Partial Hospitalization Program (PHP)	yes	no	yes	yes	yes	yes	yes	yes	yes	yes	yes	yes	yes	yes
Outpatient psychiatric care*	yes	no	N/A	no	N/A	yes	yes	yes	yes	yes	yes	yes	yes	yes
Outpatient psychotherapeutic treatment**	no	yes	yes	no	yes	yes	yes	yes	yes	no	no	no	yes	no

### Measures

We conducted problem-centered interviews [24], which allowed for a structured yet open exploration of participants’ subjective experiences related to the topic. This approach enabled us to balance the theoretical focus of the study with the flexibility to incorporate new, emergent themes during data collection.

The interview guide was developed based on the research questions of the MEN-ACCESS project. The following topics were covered in the interview guide: sociodemographics, suicide attempts, development/signs of suicidal behavior, problem-solving behavior, communication of suicidal intent, support system, protective factors, stigmatization of suicidal behavior, prevention of suicidal behavior. The interview guide can be found in the supplementary material.

### Data analysis

The interviews were audio recorded and transcribed with the transcription software f4 × [25].

Data analysis was conducted using the content structuring qualitative content analysis according to Kuckartz and Rädiker [26]. MAXQDA [27] was used for computer-assisted coding of the data.

The category system was developed both deductively and inductively. Categories were deductively drawn from the interview guide and the available evidence. The interviewer (CS) and the transcriptionists (CS, SK) created a category system separately at the beginning of the coding process. The two category systems were presented to colleagues in a qualitative research workshop and then combined into a single category system following an iterative process. In addition, categories and subcategories were adapted during the data coding process. In order to address the research question, the category system contained different types of categories: factual (objective, concretely observable facts), thematic (summarized, thematic focal points/topics), and evaluative (evaluations, subjective assessments) categories.

The first three interviews (21.4%) were coded separately by two authors (CS, JP) to ensure interrater-reliability and objectivity. Codes that did not match were discussed (by CS, JP, HG) and (sub)categories were differentiated and added in an iterative process. This process was repeated until an acceptable agreement between the coders was achieved. The final category system was then used by CS to code the remaining 11 interviews. All aspects that were coded at least 28 times across all interviews are described in detail in the results section.

The original quotations were translated into English by a native speaker.

### Researcher characteristics

The first author of the article (CS) conducted, coded and analyzed the interviews. She is a 43-year-old Western European female psychologist currently in training as a psychotherapist in psychodynamic therapy. Since 2021, she has been working in suicide research. The other team members involved in developing the category system and coding the data are also psychologists, two of them female (HG, JP) and one male (SK). The characteristics of the researchers, their preconceptions, and their potential impact on the analysis process (e.g., psychological expertise, prior therapeutic experience, clinical experience with individuals after suicide attempts), as well as their interaction with participants (e.g., gender of the interviewer), must be critically reflected upon. To address this, the authors of the present paper employed methods such as peer debriefing.

## Results

The analysis of help-seeking refers to both the initial help-seeking and renewed support during a subsequent crisis. For reasons of clarity, focus and length, only the most salient facilitating and inhibiting aspects—those coded more than 28 times across all interviews—are presented in detail. Two additional aspects, namely inhibiting “Social resources” and inhibiting “Burden of symptoms”, which were coded 24 and 25 times respectively, are not further elaborated upon in this paper. The supplementary material include the exact coding frequencies identified in the data.

The following section presents five facilitating and five inhibiting aspects that were identified. Three themes—accessibility of the support system, experiences within the support system, and psychological resources—emerged in different forms across both facilitating and inhibiting aspects. They are presented at the beginning of each respective section.

Facilitating aspects are:

- Ease of access to professional support,
- Positive experiences/ expectations regarding treatment,
- Favorable psychological resources,
- Social resources,
- Burden of symptoms.

The ease of access to professional support when seeking help (e.g., obtaining an appointment with a therapist or securing a place for treatment in a clinic) promotes the utilization of assistance. It is often a result of effective transition management between various institutions within the psychosocial support system. Many participants noted that successful transitions are typically initiated or supported by actors within the system (e.g., mental health professionals, general practitioners, psychiatrists), requiring little to no effort from the individual in need.

“After the hospital (…) I went straight into an outpatient day program, (…) and after that I was in rehab for another 8 weeks where I saw a (…) behavioral therapist. (…) I also have this worker from the addiction counseling program who told me, you can come back (to the clinic) and see me anytime, I’ll always be here to counsel you whenever you want. Do you know how comforting it is to hear that?” (P03).

For many participants, positive past experiences with the support system (e.g., positive experiences within the therapeutic relationship) and positive expectations regarding treatment outcomes (e.g., relief of symptoms, improvement in quality of life) were found to promote help-seeking — particularly when looking for renewed support in the event of a recurring crisis.

“The chemistry just works somehow with that particular psychologist. I’ve had a lot of very different psychologists over the years (…) and the most recent one is the first I’ve actually given the chance to look behind the facade and really see the whole picture so to speak.” (P07)

“I can tell that there’s an attempt to help me here. (…) I want to accept this help because I need—or want—to get along in life.” (P04).

Favorable psychological resourcesdescribed by some participants include a knowledge of mental disorders and the psychosocial support system, as well as cognitive, introspective, and communicative skills that facilitate help-seeking. This may include feeling at ease communicating with others and sharing aspects of one’s own mental health situation as well as being able to recognize and name one’s own symptoms.

“A motto I have is: ‘‘Only people who speak can be helped.’ So talking is really important, addressing problems.” (P13).

Being able to draw on social resources, such as in the form of family or friends, is another factor that promotes help-seeking. Many participants described how such informal support networks can not only provide direct support but also act as mediators, facilitating access to professional mental health services (e.g., helping with the decision to seek help, identifying support options, and scheduling appointments). It is especially helpful when family or friends have connections within the psychosocial system or personal experience with mental health issues.

“My wife is a professional in the field and she said: “You have to go to the doctor. You have to get help.” (P13).

“He (a friend) said, I should go in for an emergency consultation. Then he took me there himself and said: “Just go in, talk with them, and tell them what’s going on. After that, the rest will take care of itself. He’s been hospitalized himself (in the clinic).” (P04).

Some participants mentioned how the burden of psychological and physical symptoms, as well as impaired daily functioning, had been conducive to seeking help. The burdens included, for example, intense psychological distress (e.g., feelings of despair, low mood, anxiety) and/or physical symptoms (e.g., heart palpitations, pain). Impaired daily functioning most often referred to reduced ability to work, but also to difficulties in maintaining relationships.

“For several weeks I kept saying I don’t need a doctor, (…) it will go away on its own. And then at some point, yeah, I just felt so bad that I caved (and got help).” (P07).

Inhibiting aspects are:

- Perceived inaccessibility of professional support,
- Negative experiences/concerns regarding treatment,
- Inhibiting psychological aspects,
- Stigma, shame and guilt,
- Hegemonic masculinity norms.

Some participants expressed that the (perceived) inaccessibility of professional support at the time of need inhibits help-seeking (e.g., a perceived lack of available therapy appointments, the significant effort/burden required to access services).

“The typical wait times you always have to be seen, (…) It’s really crazy. When you’re in that situation, you don’t have two or three months.” (P01).

“The search for a therapist (…) was exhausting, making yet another call after having been turned down… no thanks.” (P14).

Many participants highlighted negative experiences with the support system (e.g., difficult therapeutic relationships, lack of symptom relief through therapies, conflicts with fellow patients) that inhibited help-seeking. Specific concerns were related to involuntary hospitalization after suicidal thoughts had been expressed. Inpatient stays on acute psychiatric wards were frequently experienced as a loss of control and autonomy.

“A feeling of helplessness, powerlessness (…) came along with not being allowed to leave, like I was in some sort of prison. (…) As soon as you’re locked up in an institution, you’re not allowed to leave of your own accord. That’s why I totally pulled myself together the first time the doctor came around (…). I gathered my concentration for half an hour beforehand and said everything was alright again. Even though I felt completely differently.” (P01).

Inhibiting psychological aspects outlined by participants refer to communication and coping strategies that negatively affect help-seeking (e.g., a preference for solving problems alone, a reluctance to address conflicts and difficulties, and alcohol/drug misuse). Further characteristics that hinder help-seeking include lower introspective ability and a lack of knowledge about mental disorders and the support system.

“I have difficulty expressing certain situations. What is going through my mind or why. It’s all the kinds of things that just can’t be fixed.” (P03).

“I would like to be able to be open about how shitty I feel. But I just can’t. It’s like some sort of block.” (P04).

Many participants described how self- and social stigma (whether feared or experienced), along with feelings of guilt and shame (e.g., related to the failure to cope with life tasks), further impede help-seeking behavior. This aspect may be fostered by masculinity norms.

“I was never able to say, ‘Help me.’ (…). That’s something you just don’t say. ‘I want to take my own life now, help me.’ It’s so humiliating you could just die on the spot.” (P12).

“Only very, very few people know about it (suicide attempt), I would never throw that in anyone’s face. I think it would stigmatize you so much. (…) That wouldn’t be good.” (P13).

Many participants stated that hegemonic masculinity norms (i.e., a man is always strong, energetic, autonomous, does not talk about feelings, provides for the family), ascribed to men by both the men themselves and by society, represent a key factor limiting help-seeking behavior.

“(…) men (…) think they have to be strong (…) you quickly start doubting yourself because you’re like: ‘Now I’m at this point where I’m weak. And when I’m weak, I’m worthless.’” (P04).

“I thought, I am a man, and a man is supposed to be able to solve his own problems by himself.” (P10).

## Discussion

The present study examines help-seeking behavior among men who have attempted suicide; however, this does not imply that the identified aspects may not also apply to women. We identified five main aspects that inhibit and five main aspects that facilitate help-seeking. The findings confirm previous research in that stigma, masculinity norms, and inhibiting psychological resources (e.g., lack of knowledge and lower introspective ability) hinder help-seeking behavior [16–18]. Negative experiences or concerns regarding treatment also emerged as inhibiting aspects in other studies [14]. At the same time, the findings highlight facilitating aspects that could be considered in gender-specific suicide prevention measures.

### Implications

Identifying these aspects provides various starting points for suicide prevention measures at the intrapersonal, interpersonal, and structural/societal levels.

At an intrapersonal level, it is important to strengthen psychological resources (e.g., knowledge, communication skills) in men and boys to promote help-seeking. Ni et al. [28] showed that, among other factors, knowledge had a positive effect on help-seeking intentions in individuals with depressive symptoms. Studies have also shown that the way individuals communicate mental health problems significantly influences access to healthcare [29]. Strengthening psychological resources can take place, among other settings, in therapeutic and educational contexts. In terms of suicide prevention, this would involve, for example, providing knowledge about mental disorders and the support system.

De-stigmatization of suicidal thoughts and behavior as well as the critical examination of masculinity norms (e.g., addressing feelings of shame and guilt, reframing “strength” and “help-seeking”) represent further core elements that should be integrated in education, suicide prevention, and medical and therapeutic interactions with men [30, 31]. These efforts are not solely the responsibility of the individual; they must also be undertaken by society. Campaigns that address both the individual man and society are recommended [20, 32]. Moreover, it is essential that gender-specific aspects of help-seeking are more thoroughly integrated into the training and continuing education of professionals within the mental health care system. A study conducted among Austrian psychotherapists suggests that gender competence is underdeveloped, with stereotypical beliefs prevailing and limited critical reflection occurring [33]. A study with medical students, on the other hand, demonstrated that curricular content during their studies can significantly enhance gender sensitivity [34].

Particularly high-risk groups, such as older men who are widowed or living alone, should receive increased attention. It may be particularly important to strengthen their social resources. In this context, the role of gatekeepers is essential. Gatekeepers, such as family members, general practitioners, and nursing staff should be sensitized and trained to recognize warning signs and provide support in seeking help [22, 35].

Furthermore, at the structural level of the help system, it is essential that help-seeking promptly leads to the actual utilization of support services, while avoiding long waiting times and patient rejections. It needs to be ensured that individuals who request help in a crisis can access support services (e.g., appointments with outpatient services or in hospitals) or are referred to appropriate support by professionals (e.g., effective transition management between institutions within the support system). Our results indicate that some men are willing to seek help, but ultimately, they do not get help because they feel that support services are not available. Other aspects that need to be acknowledged and critically examined include negative experiences within the support system and fears that deter individuals from seeking help. These negative experiences may include, for example, loss of control or autonomy, affronts by treatment staff, communication problems, or conflicts with fellow patients. It is recommended to provide opportunities for feedback during the treatment process and use complaint management within organizations in order to identify negative experiences and address conflicts effectively.

### Strengths and limitations

The present study is based on a broad range of experiences for examining help-seeking in men with a history of suicide attempts, which facilitates a deeper understanding of this under-researched topic.

Help-seeking is not a dichotomous but a complex process. A qualitative approach is therefore suitable for examining the complexities of both facilitating and inhibiting aspects. Given the limited research on this topic, an exploratory and qualitative examination of help-seeking captures both manifest aspects, which were named by participants, and latent aspects, which were interpreted by the authors.

At the same time, the qualitative approach has some limitations. To ensure the quality and trustworthiness of the analyses, the authors engaged in continuous and critical reflection on their own roles, assumptions, values, perspectives, and potential influences on the research process through collegial exchange. Peer debriefing (with a group of experts in qualitative research who were not directly involved in data collection or analysis) conducted concurrently with the analysis phase further contributed to ensuring the quality of the study. The SRQR guidelines served as a guiding framework in the development of the present paper [36].

Moreover, it must be considered that all participants had survived their suicide attempts and sought help at some point. Studies show that a large proportion of individuals who died by suicide refused to seek help [5, 37]. Therefore, the transferability of the findings to this other context may be limited. Inhibiting aspects related to help-seeking among men who died by suicide, as well as among those who do not seek any form of help, require separate investigation.

The present study focuses exclusively on the perspective of the affected men. In addition to this, their relatives as well as other stakeholders are of interest and should also be investigated. Research by Hoffmann and Wagner [38] with relatives, conducted within the MEN-ACCESS project consortium, showed that the help-seeking and communication behavior of men in suicidal crises is often ambiguous and that clarification and support are also helpful for relatives.

Although the supplementary material provides the number of coded text passages, no statements can be made about the (quantitative) significance of aspects. Additionally, the available data do not allow drawing conclusions about which aspects have an effect at particular stages in the help-seeking process (e.g., first contact or renewed support) or on specific forms of support (e.g., inpatient, outpatient). The identified aspects can be viewed as initial indications and further research is required to explore these aspects in greater depth and understand the ways in which they influence the process of help-seeking in men.

### Future research

Barriers – but especially facilitating aspects – in help-seeking among men with suicidal thoughts and behavior have not yet been extensively studied. For example, negative experiences and concerns regarding treatment could be examined in greater depth to make them available for quality assurance in the professional support system [39, 40]. While the present paper serves to provide an overview of facilitating and inhibiting aspects, a more detailed analysis could also differentiate between facilitating and inhibiting aspects relating to the initial access and those linked with sustained care in the support system.

## Conclusion

The results indicate that there are aspects that both hinder and facilitate men’s help-seeking behavior specifically among men with a history of suicide attempts. The present research focuses on key processes from which numerous clinical and structural implications can be derived. Further (qualitative) research is needed to gain a deeper understanding of these processes. To reduce the significantly elevated suicide risk among men, gender-specific aspects of help-seeking should be taken into account in suicide prevention programs.

## Supplementary Information

Supplementary Material 1.

Supplementary Material 2.

## Data Availability

The data generated and/or analyzed during the current study are not publicly available because this is not specified in the study participants’ consent forms. The data are available from the corresponding author upon reasonable request.
