# Supplementary material for: Exploring health seeking behavior among men who have attempted suicide - a qualitative study from Germany
Source: BMC Psychiatry. 2025 Sep 23;25:859. doi: 10.1186/s12888-025-07420-z (PMC12459063; doi:10.1186/s12888-025-07420-z)
Supplement: Supplementary file 1 — Supplementary Material 1. [file 12888_2025_7420_MOESM1_ESM.docx]

**Supplementary material**

Table: Coding frequencies of the facilitating and inhibiting aspects affecting help-seeking

| **Facilitating aspects** | **Number of coded segments** | **P01** | **P02** | **P03** | **P04** | **P05** | **P06** | **P07** | **P08** | **P09** | **P10** | **P11** | **P12** | **P13** | **P14** |  |
| --- | --- | --- | --- | --- | --- | --- | --- | --- | --- | --- | --- | --- | --- | --- | --- | --- |
|  |  |  |  |  |  |  |  |  |  |  |  |  |  |  |  |  |
| Ease of access to professional support | 97 | 7 | 12 | 12 | 3 | 5 | 5 | 5 | 4 | 10 | 6 | 3 | 7 | 12 | 6 |  |
| Social resources | 94 | 10 | 10 | 7 | 11 | 8 | 6 | 5 | 2 | 4 | 4 | 9 | 4 | 10 | 4 |  |
| Positive experiences/ expectations regarding treatment | 82 | 4 | 6 | 8 | 4 | 12 | 8 | 3 | 2 | 7 | 7 | 4 | 7 | 2 | 8 |  |
| Burden of symptoms | 47 | 4 | 4 | 5 | 2 | 4 | 1 | 1 | 2 | 4 | 5 | 3 | 7 | 3 | 2 |  |
| Favorable psychological resources | 47 | 5 | 3 | 5 | 1 | 2 | 3 | 2 | 2 | 5 | 3 | 2 | 1 | 7 | 6 |  |
|  | 367 |  | | | | | | | | | | | | | |  |
| **Inhibiting aspects** | **Number of coded segments** |  | | | | | | | | | | | | | |  |
|  |  |  |  |  |  |  |  |  |  |  |  |  |  |  |  |  |
| Negative experiences/ concerns regarding treatment | 100 | 2 | 1 | 7 | 8 | 6 | 17 | 8 | 9 | 7 | 4 | 6 | 5 | 7 | 13 |  |
|  |  |  |  |  |  |  |  |  |  |  |  |  |  |  |  |  |
| Stigma, shame and guilt | 74 | 7 | 3 | 1 | 10 | 2 | 1 | 7 | 7 | 7 | 3 | 8 | 5 | 10 | 3 |  |
|  |  |  |  |  |  |  |  |  |  |  |  |  |  |  |  |  |
| Inhibiting psychological aspects | 58 | 0 | 0 | 6 | 2 | 4 | 6 | 13 | 8 | 1 | 2 | 6 | 5 | 3 | 2 |  |
| Perceived inaccessibility of professional support | 57 | 4 | 3 | 8 | 2 | 8 | 4 | 5 | 10 | 1 | 1 | 4 | 5 | 0 | 2 |  |
| Hegemonic masculinity norms | 33 | 2 | 3 | 3 | 4 | 0 | 0 | 1 | 5 | 3 | 2 | 2 | 5 | 1 | 2 |  |
| Social resources | 25 | 0 | 2 | 0 | 3 | 1 | 2 | 2 | 3 | 3 | 3 | 0 | 3 | 2 | 1 |  |
| Burden of symptoms | 24 | 0 | 0 | 2 | 0 | 0 | 2 | 2 | 4 | 2 | 0 | 0 | 0 | 5 | 2 |  |
|  | 371 |  | | | | | | | | | | | | | |  |
