# Supplementary material for: Exploring health seeking behavior among men who have attempted suicide - a qualitative study from Germany
Source: BMC Psychiatry. 2025 Sep 23;25:859. doi: 10.1186/s12888-025-07420-z (PMC12459063; doi:10.1186/s12888-025-07420-z)
Supplement: Supplementary file 2 — Supplementary Material 2. [file 12888_2025_7420_MOESM2_ESM.docx]

**Supplementary material**

**Interview Guide**

**Introduction to the Interview**

As I have explained, I will now ask you some questions related to your suicide attempt(s) or experiences with suicidal thoughts, as well as the help you have sought or barriers you have faced in seeking help, and the communication of your suicidal intent. It is also important for us to understand what you consider especially important for men regarding suicide prevention.

We will record this interview using this audio recorder. To protect your personal data, all information you provide will be anonymized, treated with strict confidentiality, and handled according to data protection regulations.

In this interview, your personal experiences, insights, and opinions are important to me. I will ask you to respond as fully as possible to the different topics. I may occasionally take notes. We can pause or stop the interview at any time. You can also end the interview at any time without providing a reason. The entire interview should last no longer than two hours, with regular breaks (during which we will also ventilate the room).

If you agree with this procedure and have no further questions, I would like to begin.

**Sociodemographic Data**

- How old are you?
- What is your marital status? (single, in a committed relationship, married, divorced/separated, widowed)
- Do you have children? If yes, how many?
- Do you live alone or with others?
- What is your highest educational qualification? What is your current occupation?
- If German is not your native language, how would you rate your German skills? (very good – none)
- Have you experienced any significant life events in the last two years (e.g., breakup, job loss, both, none)?
- Do you take any regular medication? If yes, which ones?
- Have you been diagnosed with a mental health disorder? If yes, do you know which one?

**Suicide Attempts**

- Have you ever attempted to take your own life with the intent to die?
- How many attempts have you made?
- How old were you at your first attempt?
- When was your most recent suicide attempt?
- What method did you use?
- On average, how long did you think about suicide before attempting it?

**Development/Signs**

- How did the suicide attempt come about?
- What thoughts, feelings, or behaviors preceded the suicide attempt?
- What changes might others have noticed?
- How did you explain your situation to yourself?

**Problem-Solving Behavior**

- What problems or circumstances contributed to the suicide attempt?
- How did you try to cope with these problems or circumstances before the attempt?
- How have you previously dealt with difficult situations (e.g., conflicts with family)?

**Communication**

- Did you talk to anyone about your suicidal thoughts or situation?
- Did you share your suicidal thoughts or situation in other ways (e.g., social media, letters)?
- If yes: To whom did you disclose? Were there close people you did not tell, and why?
- What did you share, and what was the reaction?
- If no: Why did you not talk to anyone?
- Could someone else have noticed your situation in other ways (e.g., certain behaviors)? How might they have noticed?

**Help System/Protective Factors**

- Who are important supportive persons for you?
- What other protective factors do you have (activities/hobbies, experiences, attitudes that help)?
- Did you seek help or support?
- If yes: Where or from whom did you seek help/support?
- If no: Why did you not seek support?
- Where do you think people with suicidal thoughts or in crisis can get help/support?
- What are your thoughts about the psychological/psychiatric care system?
- What are your thoughts about men who seek psychological/psychiatric help?

**Stigma**

- What do you think is the general opinion about men who have suicidal thoughts or have attempted suicide?
- What do you think is the general opinion about men who seek psychological or psychiatric help?

**Prevention**

- What do you think men who have suicidal thoughts or consider suicide need?
- What, in your view, can help specifically men when suicide seems like the only way out?
- What would you have wished for from people around you when you were feeling worse?
- When it comes to help for suicidal thoughts, who might it help to talk to (e.g., professionals, other affected individuals)? Who would you have spoken to?
- What experience or advice would you pass on to other men?
- What information would have been helpful for you?
- More generally: What should an offer for men include to prevent suicides? Or: What do you think is important to prevent suicides in men? Or: What do you think is important to help men with suicidal thoughts?
